# Elasticity and cross-sectional thickness of paraspinal muscles in progressive adolescent idiopathic scoliosis

**DOI:** 10.3389/fped.2024.1323756

**Published:** 2024-03-07

**Authors:** Yunli Fan, Haiping Zheng, Lin Feng, Michael K. T. To, Guan-Ming Kuang, Eric H. K. Yeung, Kenneth M. C. Cheung, Li Liu, Jason P. Y. Cheung

**Affiliations:** ^1^ Department of Physiotherapy, The University of Hong Kong—Shenzhen Hospital, Shenzhen, Guangdong, China; ^2^ Department of Orthopaedics and Traumatology, The University of Hong Kong, Hong Kong, Hong Kong SAR, China; ^3^ Department of Medical Imaging—Ultrasound Division, The University of Hong Kong—Shenzhen Hospital, Shenzhen, Guangdong, China; ^4^ Department of Orthopaedics and Traumatology, The University of Hong Kong—Shenzhen Hospital, Shenzhen, Guangdong, China

**Keywords:** adolescent idiopathic scoliosis, elasticity, cross-sectional thickness, shear wave speed, curve progression

## Abstract

**Objectives:**

(1) Compare the cross-sectional thickness (CST) and shear wave speed (SWS) of paraspinal muscles (PSM) in adolescent idiopathic scoliosis (AIS) with and without curve progression; (2) investigate the relationship between CST/SWS and radiographic characteristics in AIS with curve progression; (3) compare the CST/SWS between AIS and non-scoliosis controls.

**Methods:**

This cross-sectional study analyzed the CST and SWS of PSM in 48 AIS with mild to moderate curvature and 24 non-scoliosis participants. Participants with scoliosis greater than 45° of Cobb angles were excluded. The Change of Cobb angles within the last 6-months was retrieved to allocate AIS into progression and non-progression groups. The SWS and CST of multifidus; longissimus and iliocostalis of the major curve were measured using B-mode ultrasound image with an elastography mode. Discrepancies of the SWS (SWS-ratio: SWS on the convex side divided by SWS on the concave side) and CST (CST-ratio: CST on the convex side divided by CST on the concave side) at the upper/lower end and apical vertebrae were studied.

**Results:**

A higher SWS at the apical vertebrae on the concave side of the major curve (multifidus: 3.9 ± 1.0 m/s vs. 3.1 ± 0.6 m/s; *p *< 0.01, longissimus: 3.3 ± 1.0 m/s vs. 3.0 ± 0.9 m/s; *p *< 0.01, iliocostalis: 2.8 ± 1.0 m/s vs. 2.5 ± 0.8 m/s; *p *< 0.01) was observed in AIS with curve progression. A lower SWS-ratio at apical vertebrae was detected with a greater vertebral rotation in participants with curve progression (multifidus [grade II]: 0.7 ± 0.1 vs. grade I: 0.9 ± 0.2; *p *= 0.03, longissimus [grade II]: 0.8 ± 0.2 vs. grade I: 1.1 ± 0.2; *p *< 0.01). CST was not different among the progressive, non-progressive AIS and non-scoliosis controls.

**Conclusions:**

Increased SWS of PSM without change of CST was observed on the concave side of the major curve in participants with progressive AIS.

## Introduction

Adolescent idiopathic scoliosis (AIS), the most common spine deformity afflicting up to 4% of children globally, refers to a lateral spinal curvature with a Cobb angle larger than 10° coupled with vertebral deformation and sagittal hypokyphosis (1). A recent population-based screening program in China showed 14% incidence of scoliosis in females aged 13–14 years (2). The rapid increase in scoliosis incidence may be due to lifestyle changes, as children spend less time exercising but more time using electronic devices and social media. Without early detection of curve progression, scoliosis can be easily under-diagnosed, as clinically 15%–30% of scoliotic patients will experience curve progression during pubertal growth, leading to Cobb angles larger than 45° for surgical intervention (1). Biomechanical studies of AIS with operative curve magnitude have identified three alterations in muscle architecture in addition to vertebral deformity: increased fat infiltration (3), asymmetrical muscle volume (4), and paraspinal muscle (PSM) stiffness (5). The mechanisms underlying these characteristic changes remain unclear, but evidence suggests that increased muscle stiffness and atrophy are weakening muscle contraction, and change leads to asymmetrical muscle activities, potentially contributing to scoliosis progression (6).

Electromyography (EMG) and magnetic resonance imaging (MRI) have been used to assess motor control (7, 8), in terms of static and dynamic muscle contraction in different postures, and the morphology of PSM in AIS (4). Specifically, higher fat infiltration and muscle atrophy have been observed in the paraspinal muscles at the apical vertebrae (AV) of the major curve with Cobb angle of 50° or more (3, 4). Studies have also revealed higher EMG activity at curve convexity, which was correlated with a greater side deviation (SD) of AV (8, 9). Those findings suggest that the biomechanical properties of PSM and vertebral deformity are interrelated. However, MRI and EMG are not standard clinical methods for patients with AIS. Shear wave elastography, as an alternative technique, stimulates tissue vibration and generates shear wave through the emission of acoustic radiation force (10). The shear wave speed (SWS) is then calculated and used to evaluate the biomechanical property of musculoskeletal tissues (10). The harder tissue is, the faster SWS will be (11, 12). Ultrasonography has been preferred in studies to evaluate the biomechanical features of PSM, uncovering an asymmetry in stiffness and thickness of PSM in AIS with operative curve magnitude (13–15). In cases of mild to moderate scoliosis, this relationship remains inconclusive. No study has examined how various paraspinal muscle characteristics are correlated with temporal changes in the scoliotic curvature during curve progression in patients with mild to moderate AIS. Accordingly, evaluating paraspinal muscle properties, along with known prognostic factors such as spinal growth velocity, may enhance the prediction of subsequent curve progression (6). Identifying muscle property changes of PSM in mild to moderate AIS during the progressive period can offer clinical value by aiding early treatment decisions, and by aiming to prevent curve progression into the operative threshold.

This study used real-time ultrasonography to assess the cross-sectional thickness (CST) and muscle elasticity, interpreted using the SWS, of multifidus, longissimus, and iliocostalis muscles at AV and upper end vertebrae (UEV) and lower end vertebrae (LEV) of the major curve. Our findings elucidate the characteristics of biomechanical properties of PSM in mild to moderate AIS during curve progression. In this study, we posed three specific research questions:

(1) How do the CST and elasticity of PSM in AIS differ in cases with and without curve progression?
(2) How are the biomechanical features of PSM and vertebral deformities (Cobb angle, apical wedging [AW]/rotation [AR]/SD, thoracic kyphosis [TK] and lumbar lordosis [LL]) correlated with curve progression?
(3) How do CST and the elasticity of PSM differ between AIS and non-scoliosis controls?

## Materials and methods

This was a cross sectional study conducted in accordance with the Declaration of Helsinki principles. Ethics approval [(2022)123] was obtained from the local institutional review board, and informed consent was obtained from the participants and their parents/legal custodians before study.

## Participants

Participants with AIS and non-scoliosis controls were consecutively enrolled into this study from June 2022 and July 2023 in our spine clinic. The inclusion criteria were as follows:

(1) Diagnosis of AIS, with Cobb angle ranging from 10° to 45°
(2) Latest radiography taken no more than one month before this study
(3) Prior radiography was within the last 6 months showing changes in Cobb angles
(4) Non-scoliosis controls with Cobb angle less than 10°
(5) Age ranging from 10 to 17 years
(6) Skeletal immaturity characterized by Risser stage of less than 5.

The exclusion criteria were as follows:

(1) Diagnoses other than AIS
(2) Cobb angle greater than 45°
(3) Presence of back pain [this criteria was set to minimize the effects of pain in altering the architecture of the multifidus (16)]
(4) A history of spinal orthosis [this criteria was set to minimize the effects of bracing on muscle weakness (9), which affects the biomechanical properties of back muscles].

### Radiographic measurement

Two spine surgeons retrieved and measured the latest and preceding 6-months radiographic parameters of the participants. These included the curve pattern, Cobb angle of the major curve, AW, SD, AR, TK (T5-12), LL (L1-5), and Risser stage. AR was measured at the major curve AV using the Nash and Moe system, graded from I to V (17). SD was defined as the distance between the center of AV and the central sacral vertical line (18). AW was calculated as the ratio of the major curve AV between sides $\left(\mathrm{AW}=\frac{\mathrm{vertebral}\;\mathrm{height}\;\mathrm{on}\;\mathrm{convex}\;\mathrm{side}}{\mathrm{vertebral}\;\mathrm{height}\;\mathrm{on}\;\mathrm{concave}\;\mathrm{side}}\right)$ reflecting the reformation of the AV in the coronal plane (19). Skeletal immaturity was assessed in terms of Risser staging, with 0 referring to Risser sign 0 with open triradiate cartilage, 0 + referring to Risser sign 0 with closed triradiate cartilage and 4 + indicating a stage where the iliac apophysis is capped but not yet fused (20). Additionally, Lonstein-Carlson risk of scoliosis progression $\left(\mathrm{LCR}=\frac{\mathrm{Cobb}\;\mathrm{angle}-3*\;\mathrm{Risser}\;\mathrm{sign}}{\mathrm{Chonological}\;\mathrm{age}}\right)$ was adopted to evaluate participants' risk of prospective curve progression (21). Using the LCR equation in addition to the changes of Cobb angles, which helped in discriminating progressive and non-progressive cases before group allocation (22). Curves were classified as either major thoracic curves (major T: single right thoracic or a major right thoracic with a minor left lumbar) or major lumbar curves (major L: single left lumbar or a major left lumbar with a minor right thoracic or left thoracolumbar curve). Participants were allocated into groups based on the increase of curve magnitude:

(1) Those with a change greater than 5° to the progression group
(2) Those with a change of Cobb angle between −5° and 5° were allocated to the non-progression group
(3) Those with a Cobb angle less than 10° were allocated to the non-scoliosis group.

## Ultrasonographic measurement

The elasticity and CST of the multifidus, longissimus, and iliocostalis muscles was assessed using B-mode ultrasound imaging with elastography. The elasticity index was quantified by measuring the SWS in this study. European Federation of Societies for Ultrasound in Medicine and Biology (EFSUMB) recommended using SWS to evaluate biomechanics in musculoskeletal system (23). SWS was chosen for its superior reliability and repeatability in assessing the elasticity of skeletal muscles, which have anisotropic and inhomogeneous tensile properties (24). Thus, SWS was determined using force-deformation data between the superficial muscle layer and bottom muscle layer (longissimus and iliocostalis)/the bone surface of vertebral laminae (multifidus) at the UEV, AV, and LEV for participants with scoliosis, and at the eighth thoracic (T8) and the third lumbar vertebrae (L3) for non-scoliosis participants, using ultrasound elastography (5).

Ultrasonographic measurement was performed with participants in a prone lying posture on a physiotherapy plinth, with a small pillow situated beneath the abdomen to minimize lumbar spine movement. A spinal surgeon located the spinal process of AV, UEV, LEV, T8, and L3 by palpation according to their radiographs and marked the skin before ultrasonographic measurement. The location of each targeted vertebra and the adjacent multifidus, longissimus, and iliocostalis muscles were identified with a longitudinal sonographic scan before data collection. To acquire the CST and SWS of paraspinal muscles, a real-time diagnostic high-definition ultrasound unit (HDI-5000, Resona 7OB, Mindrary, Shenzhen, China) with a shear wave elastography mode, was used, along with a 3–9 MHz transducer (L9-3). With a posterior approach, the transducer was held perpendicular to the skin surface on the back of the patient. Cross-sectional images of the paraspinal muscles, 3 cm horizontally away from the spinal process, on both sides of the spine were acquired, with the echogenic tip of the spinous process in the middle and the vertebral laminae at the anterior margin of the multifidus muscles serving as consistent landmarks. The maximum anteroposterior diameter of the multifidus, longissimus, and iliocostalis muscles were collected at AV, UEV, and LEV levels in scoliotic participants and at T8 and L3 in non-scoliosis controls. A loading force of 4–5 N was then applied to the paraspinal muscles through the elastography transducer and subsequently released. Loading–unloading cycles were performed at least three times to derive the effective elastography (image with reliability ≥0.9). The load-indentation response data were based on the applied load and the deformation ratio. The ultrasound scanning was repeated five times, yielding five images for determining the average values of the CST and SWS. Both SWS ratio $\left(\mathrm{SWS}\text{-}\mathrm{ratio}=\frac{\mathrm{sws}\;\mathrm{on}\;\mathrm{the}\;\mathrm{convex}\;\mathrm{side}}{\mathrm{sws}\;\mathrm{on}\;\mathrm{the}\;\mathrm{concave}\;\mathrm{side}}\right)$ and the ratio of cross-sectional thickness $\left(\mathrm{CST}\text{-}\mathrm{ratio}\;=\;\frac{\mathrm{CST}\;\mathrm{on}\;\mathrm{the}\;\mathrm{convex}\;\mathrm{side}}{\mathrm{CST}\;\mathrm{on}\;\mathrm{the}\;\mathrm{concave}\;\mathrm{side}}\right)$ were calculated to interpret the discrepancies in muscle elasticity and thickness of the PSM in AIS. The SWS-ratio and CST-ratio were determined by the SWS and CST on the right (left) side at T8 (L3) divided by the SWS and CST on the left (right) side at T8 (L3) for non-scoliosis controls. This was to match the calculations regarding the curve pattern of participants with AIS.

### Reliability tests and power analysis

The reliability tests involved four AIS and two non-scoliosis participants, with two operators participating. The SWS and CST were measured by each operator five times at 3 cm horizontally away from spinal process of AV on the convex and concave sides of the major curve in participants with AIS, and at the T8 and L3 vertebrae in non-scoliosis controls. These repeated measurements demonstrated strong interoperator reliability for both SWS (multifidus intraclass coefficient [ICC_3,2_]: 0.90, longissimus [ICC_3,2_]: 0.92, iliocostalis [ICC_3,2_]: 0.90) and CST (multifidus [ICC_3,2_]: 0.89, longissimus [ICC_3,2_]: 0.93, iliocostalis [ICC_3,2_]: 0.88) and consistent intraoperator repeatability across consecutive trials for SWS (multifidus: coefficient of variation [CV] 1.4%–2.3%, longissimus: [CV] 1.1%–2.0%, iliocostalis: [CV] 1.8%–2.1%) and CST (multifidus: [CV] 3.1%–4.0%, longissimus: [CV] 2.9%–3.6%, iliocostalis: [CV] 2.4%–4.1%).

A power analysis was conducted to compare AIS and non-scoliosis groups. Our pilot SWS data set on four AIS and two non-scoliosis participants revealed a mean difference of 0.22 of SWS-ratio at the AV, with the largest standard deviation being 0.37. Power calculations indicated that 60 participants were required to achieve a power of 80% at *p *= 0.05, assuming a null hypothesis of no difference.

### Statistical analysis

Quantitative variables, namely age, body mass index (BMI), sport intensity, asymmetrical trunk rotation (ATR), Cobb angles, SWS, CST, AW, SD, TK, and LL, were compared using one-way analysis of variance (ANOVA). Categorical variables, namely sex, handedness, AR, Risser stage, and curve patterns, were tested using Pearson's Chi-squared tests. The *t* test was used to study the difference in SWS and CST between the curve convexity and concavity in participants with AIS. Post hoc intra-/inter-group comparison and multivariate general linear model analysis were performed to investigate the relationship between SWS/CST and radiographic parameters in participants with curve progression if a significant difference in either SWS-ratio or CST-ratio was detected between groups. All statistical analyses were performed using SPSS 27.0 for Windows (SPSS, Chicago, IL, USA).

## Results

### Patient characteristics

**Figure 1:**
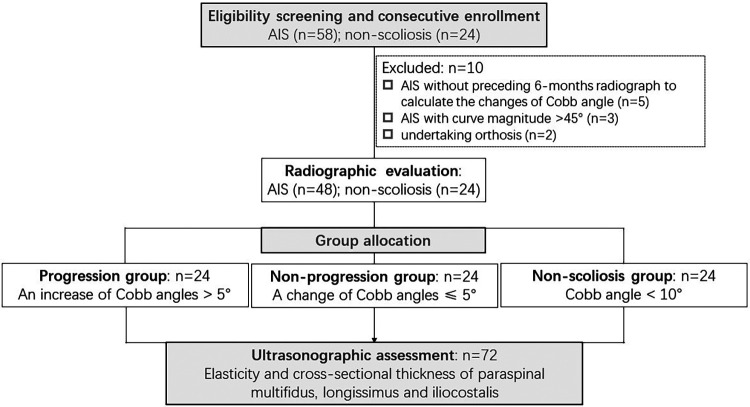
Study flow chart. AIS, adolescent idiopathic scoliosis.

After curve magnitudes in 144 spinal radiographs from 58 adolescents with scoliosis and 24 peers without scoliosis were measured, 48 participants with AIS (progression [*n* = 24]: increase of Cobb angle by 6.2 ± 1.8°; LCR = 2 ± 0.8 [80%], non-progression [*n* = 24]: change of Cobb angle ≤0.0 ± 2.3°; LCR = 1 ± 0.8 [<20%]) and 24 non-scoliosis controls [Cobb angle: 8.0 ± 1.0° with LCR = 0.2 ± 0.4 (<5%)] were enrolled in this study (Figure 1). The groups did not significantly differ in age, sex, BMI, handedness, Risser stage, curve pattern or sport intensity (Table 1). The greater ATR, AR, SD and curve magnitude and the lower TK were observed in participants with progressive AIS (Table 1). Upon comparison between latest radiographs and those 6 months prior, increases in AR (latest radiograph: grade II [*n* = 10]; grade I [*n* = 14], preceding radiograph: grade II [*n* = 5]; grade I [*n* = 19], *χ*^2^ test: *p *< 0.01) and SD (38 ± 9.2 mm vs. 42 ± 9.9 mm, paired *t* test: *p *< 0.01) were detected, along with an increase of Cobb angles in the progression group.

**Table 1:** Participants’ characteristics.

	AIS		Non-scoliosis controls (*n* = 24)	*P* value
	Progression (*n* = 24)	Non-progression (*n* = 24)		
Age: years	12 ± 2.2	13 ± 1.9	12 ± 2.1	0.1
Sex (female/male): *n*	15/9	19/5	17/7	0.4
BMI	18 ± 1.9	18 ± 3.6	19 ± 2.4	0.06
Handedness (right/left): *n*	21/3	20/4	20/4	0.9
Sport intensity: hours/week	7 ± 1.2	7 ± 1.2	7 ± 1.4	0.3
ATR: °	7 ± 1.2	6 ± 1.4	5 ± 0.7	<0.01
LCR	2 ± 0.8 (80%)	1 ± 0.8 (<20%)	0.2 ± 0.4 (<5%)	<0.01
Risser staging: *n*				0.2
0	3	0	5	
0+	2	0	2	
1	4	4	2	
2	6	5	5	
3	0	5	3	
4	8	8	4	
4+	1	2	3	
Curve pattern: *n*				0.8
**Major T** (T4–12)/AV at T8/9	12	11	–	
**Major L** (T10–L5)/AV at L3	8	10	–	
**Major TL** (T8–L3)/AV at T12/L1	4	3	–	
Cobb angle: °				
Present	32 ± 6.8*	26 ± 9.1	8 ± 1.0	<0.01
Preceding 6-months	26 ± 6.2	26 ± 8.7	–	0.95
Changes	6.2 ± 1.8	0.0 ± 2.3	–	<0.01
Vertebral deformity				
AR (AR-prior): *n*	II = 10, I = 14	II = 4, I = 20	II = 0, I = 24	0.02
	(II = 5, I = 19)	(II = 4, I = 20)	(II = 0, I = 24)	(0.2)
SD (SD-prior): mm	42 ± 9.9* (38 ± 9.2)	32 ± 11.5 (33 ± 11)	–	<0.01
AW (AW-prior)	1.1 ± 0.1 (1.1 ± 0.1)	1.1 ± 0.1 (1.1 ± 0.1)	–	0.1
TK (TK-prior): °	19 ± 2.3° (18 ± 3.3°)	24 ± 4.8° (23 ± 3.9°)	–	<0.01
LL (LL-prior): °	38 ± 3.3° (39 ± 3.5°)	40 ± 2.9° (40 ± 3.2°)	–	0.3

BMI, body mass index; ATR, asymmetrical trunk rotation; LCR, Lostein-Carlson risk of scoliosis progression; Major T, a major thoracic scoliosis; Major L, a major lumbar scoliosis; Major TL, a major thoracolumbar scoliosis; AV, apical vertebrae; AR, apical rotation; SD, side deviation; AW, apical wedging; TK, thoracic kyphosis; LL, lumbar lordosis.

* Significantly differed to the value at preceding 6-months in the progression group.

### SWS and CST

#### Progression vs. non-progression

**Figure 2:**
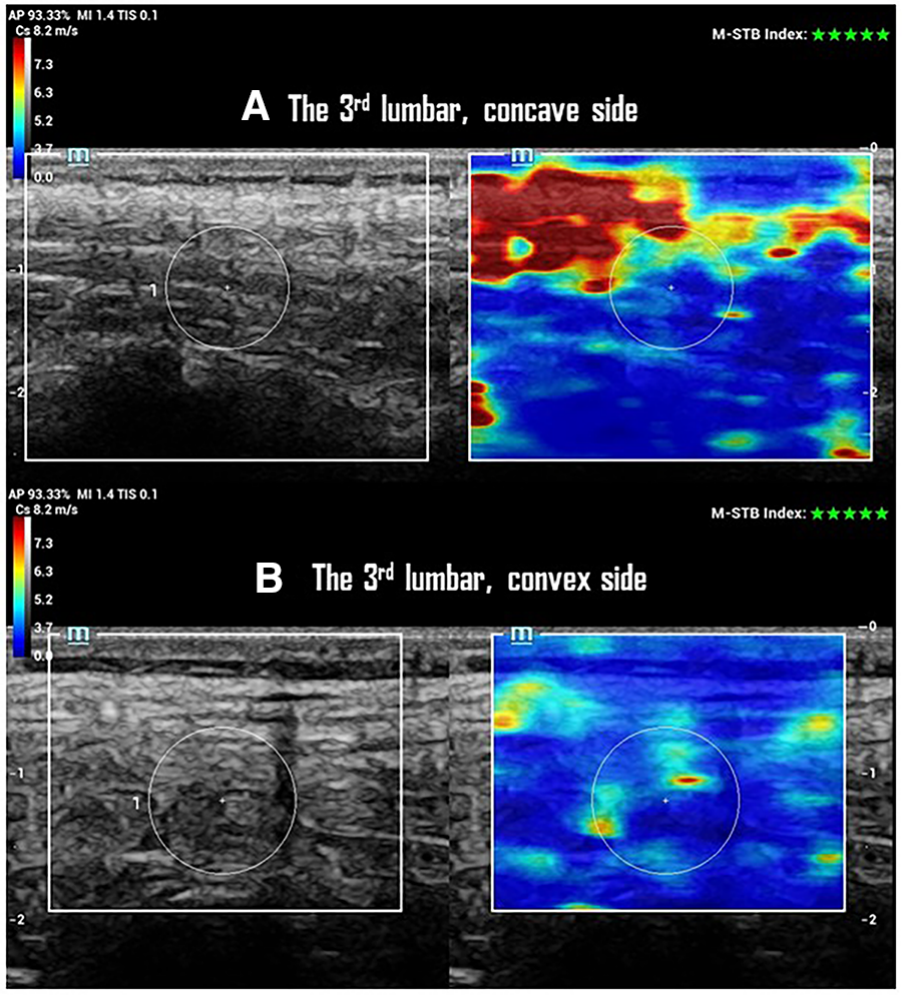
SWS and CTS of paraspinal multifidus at the 3rd lumbar vertebrae of one participant with a 25° left lumbar scoliosis in the progression group. (**A**) Ultrasonographic imaging shows a 10 mm CTS with 4.4 ± 1.4 m/s of SWS in multifidus on the concave side of the curve. (**B**) Ultrasonographic imaging shows a 10 mm of CTS with 3.9 ± 1.0 m/s of SWS in multifidus on the convex side of the curve.

A higher SWS on the concave side with no change of CST was observed at AV of the major curve in the progressive case (Figure 2). This resulted in a smaller SWS-ratio (repeated measurements: 0.8 ± 0.2, 0.9 ± 0.3, 0.9 ± 0.2) at the AV of the multifidus (*p *< 0.01), longissimus (*p *< 0.01), and iliocostalis (*p *= 0.04) muscles in the progression group (Table 2). However, these findings were not observed at the UEV or LEV in either progression or non-progression groups. The CST and CST-ratio did not differ between the progression and non-progression groups at AV, UEV, or LEV (Table 2). The CST showed no differences between scoliosis and non-scoliosis controls.

**Table 2:** SWS and CTS of PSM.

	AIS						Non-scoliosis controls (*n* = 24)		
	Progression (*n* = 24)			Non-progression (*n* = 24)					
	Multifidus	Longissimus	Iliocostalis	Multifidus	Longissimus	Iliocostalis	Multifidus	Longissimus	Iliocostalis
SWS (convex; concave)									
UEV: m/s	3.5 ± 1.0;	3.1 ± 1.3;	2.7 ± 0.7;	3.4 ± 1.2;	3.1 ± 1.0	2.6 ± 0.6;			
	3.5 ± 1.0	3.1 ± 0.9	2.7 ± 0.8	3.4 ± 1.0	2.9 ± 0.8	2.6 ± 0.7			
AV: m/s	3.1 ± 0.6**^,^*;	3.0 ± 0.9**^,^*;	2.5 ± 0.8**;	3.6 ± 1.2;	3.3 ± 1.2;	2.6 ± 0.8;	3.1 ± 0.8;	2.8 ± 0.6;	2.6 ± 0.6;
	3.9 ± 1.0*	3.3 ± 1.0*	2.8 ± 1.0	3.4 ± 1.1	2.8 ± 0.6	2.5 ± 0.7	3.1 ± 0.8	2.8 ± 0.7	2.5 ± 0.5
LEV: m/s	3.2 ± 0.9;	3.2 ± 0.8;	2.7 ± 0.7;	3.3 ± 0.9;	3.2 ± 1.4;	2.7 ± 0.9;			
	3.2 ± 0.8	3.0 ± 0.8	2.8 ± 0.7	3.2 ± 1.0	2.7 ± 0.5	2.5 ± 0.5			
SWS-ratio									
UEV	1.0 ± 0.2	1.0 ± 0.2	1.0 ± 0.3	1.0 ± 0.3	1.0 ± 0.3	1.1 ± 0.2			
AV	0.8 ± 0.2*	0.9 ± 0.3*	0.9 ± 0.2*	1.1 ± 0.3	1.2 ± 0.3	1.1 ± 0.3	1.1 ± 0.2	1.0 ± 0.2	1.0 ± 0.2
LEV	1.1 ± 0.2	1.1 ± 0.2	1.0 ± 0.2	1.0 ± 0.2	1.2 ± 0.4	1.1 ± 0.5			
CST (convex; concave)									
UEV: mm	9.9 ± 2.2;	9.8 ± 2.6;	8.5 ± 2.8;	9.3 ± 2.2;	10.8 ± 3.5;	9.2 ± 2.6;			
	9.0 ± 1.9	10.0 ± 3.4	8.1 ± 2.5	8.9 ± 2.4	10.5 ± 3.0	8.7 ± 2.5			
AV: mm	8.4 ± 1.9;	13.1 ± 2.9;	10.1 ± 3.3;	8.7 ± 1.8;	12.7 ± 4.3;	11.4 ± 3.9;	8.4 ± 1.9;	13.7 ± 4.9;	12.0 ± 6.2;
	8.3 ± 1.7	12.7 ± 3.0	11.1 ± 2.8	8.6 ± 2.4	12.5 ± 4.1	11.1 ± 3.1	8.2 ± 2.2	13.1 ± 4.4	11.9 ± 5.5
LEV: mm	8.2 ± 2.0;	14.6 ± 3.6	13.3 ± 4.1;	8.0 ± 2.5;	14.1 ± 4.8	13.4 ± 4.1;			
	7.8 ± 2.0	14.3 ± 3.6	14.5 ± 4.0	7.8 ± 1.9	13.8 ± 4.8	12.8 ± 4.3			
CST-ratio									
UEV	1.0 ± 0.1	1.0 ± 0.2	1.1 ± 0.2	1.3 ± 0.2	1.0 ± 0.2	1.1 ± 0.2			
AV	1.0 ± 0.2	1.0 ± 0.2	1.0 ± 0.2	1.0 ± 0.2	1.0 ± 0.2	1.1 ± 0.2	1.0 ± 0.1	1.0 ± 0.1	1.0 ± 0.2
LEV	1.1 ± 0.2	1.0 ± 0.1	0.9 ± 0.2	1.0 ± 0.2	1.0 ± 0.2	1.0 ± 0.2			

AIS, adolescent idiopathic scoliosis; SWS, shear weave speed (m/s). CST, cross-sectional thickness. UEV, upper end vertebrae. AV, apical vertebrae (measured at the 8^th^ thoracic and the 3^rd^ lumbar vertebral levels in the non-scoliosis group). LEV, lower end vertebrae.

* significantly differ to the non-progression group and non-scoliosis controls

** Significantly differ to the concave side in the progression group.

A discrepancy in SWS, characterized by a smaller SWS-ratio at AV, was observed in the progression group but not in the non-scoliosis controls (repeated measurements: multifidus [*F* = 8, *p *< 0.01], longissimus [*F* = 6.8, *p *< 0.01], iliocostalis [*F* = 3.8, *p *= 0.03], Table 2). Moreover, CTS was consistent between sides in both AIS and non-scoliosis controls at all tested vertebral levels (Table 2).

#### Correlation with clinical features

Significant differences in ATR, AR, SD, and TK were observed between the progression and non-progression groups (Table 1). The lower SWS-ratio at AV of multifidus (grade II: 0.7 ± 0.1 vs. grade I: 0.9 ± 0.2; *p *= 0.03) and longissimus (grade II: 0.8 ± 0.2 vs. grade I: 1.1 ± 0.2; *p *< 0.01) were detected in progressive cases with higher AR. The lower SWS-ratio was weakly related to the greater curve magnitude and ATR in participants with AIS (linear regression analysis: latest Cobb angles [*r* = 0.3, *p *= 0.04], ATR [*r* = 0.4, *p *= 0.03]). However, no such relationship was detected between the SWS ratio and SD/TK. The smaller SWS-ratio at AV was distinctly found in multifidus of male participants with curve progression (0.8 ± 0.1 vs. 1.0 ± 0.3, *p *< 0.01).

## Discussion

The data acquired in this study addressed our second research question, revealing that curve progression in mild to moderate scoliosis is associated with distinct changes in the elasticity of PSM. This association was observed with greater AR of the major curve, independent of the curve pattern and SD/KT/LL. However, no such relationship was detected in the CST of PSM. Moreover, no difference in CST of PSM was found between the non-scoliosis and AIS groups in this study cohort (Table 2). Overall, the multifidus, longissimus, and iliocostalis muscles on the concave side of the major curve exhibited greater stiffness (a higher SWS) without a change in thickness in participants with mild to moderate progressive AIS (Figure 2). This change may lead to a discrepancy in PSM elasticity, potentially affecting muscle contractile function.

A difference in muscle contractile activity is considered as a risk factor for scoliosis progression, along with skeletal immaturity and vertebral deformity (6). The observed changes in muscle elasticity without muscle thickness alterations suggest that stiffness and thickness of PSM do not occur simultaneously in mild to moderate scoliosis during curve progression. The increased muscle elasticity leads to a stiffer muscle architecture (24). A significant decrease in muscle thickness and an increase in muscle stiffness, predominantly on the concave side, were observed in patients with operative scoliotic magnitude (4, 5, 12, 15, 16). Such a correlation was not observed in our study, likely due to the inclusion of participants with only mild to moderate scoliosis. This implies that changes in muscle elasticity precede changes in muscle volume during scoliosis progression. Given that muscle stiffness weakens muscle contractile function and may affect the spinal stability (16), our findings imply that the asymmetrical elasticity of PSM is a potential driving force and being a predictive marker in scoliosis progression. Thus, early intervention such as scoliosis-specific exercise to improve the discrepancy in muscle activity may prevent curve progression and reduce muscle atrophy in patients with AIS (25).

A higher elasticity in multifidus, longissimus, and iliocostalis muscles was observed on the concave side of the major curve at AV but not at UEV/LEV in the progression group. Additionally, this discrepancy in muscle elasticity was distinctive in progressive cases with greater AR and curve progression. These findings confirm that changes in biomechanical properties and vertebral deformity are interrelated. AIS, being the most common structural scoliosis, often results in the AV being most deformed in rotation (17). Our findings are consistent with those of studies indicating increased muscle stiffness predominantly on the concave side at the apical level (3–5, 13, 15), suggesting a relationship between muscle stiffness and vertebral rotation. Alternatively, the lower SWS-ratio was weakly related to greater ATR and curve magnitude in this study cohort. This may be because of the difference of ATR was <1° between the progression and non-progression groups in this study, and curve magnitude was reduced simultaneously with lying position when doing ultrasound measurement. The 1° of difference was statistically different but within the measurement error. Other researchers have identified a stiffer muscle architecture associated with greater SD in AIS (5). However, such a relationship was not observed in our study, possibly due to the smaller curve magnitude and lesser SD in our study cohort. The decrease in SD is associated with a decrease in Cobb angles when the spine is not bearing weight (26), a condition referred to as gravity unloaded, such as in the prone lying posture used for elastography in our study. In addition, a smaller SWS-ratio, indicative of higher muscle elasticity on the concave side, was found in male participants with curve progression. Possible explanations include differences between the sexes in the muscle architecture of back muscles (27) and a higher likelihood of progression in male AIS (1). Our previous study found that weaker EMG activity on the concave side of the major curve was related to curve progression in untreated AIS (8). This study expands on that work, suggesting that the muscle is stiffer on the concave side of the major curve in progressive mild to moderate AIS. Thus, interventions targeting the reduction of PSM stiffness on the concave side of the major curve merit future study.

The CST of PSM did not differ between the AIS group and the non-scoliosis controls in this study cohort. This finding contrasts with another study that detected a smaller CST on the concave side of the major curve in patients with AIS with operative curve magnitude (15). The smaller CST was attributed to muscle atrophy, which was caused by the inhibition of muscle activity due to muscle stiffness and the inability of a muscle or ligament to return to its original resting length, owing to increased fat infiltration in PSM (3). This smaller cross-sectional area, in conjunction with a larger fat area in PSM in AIS with big curve magnitude, implies increased infiltration of noncontractile materials and a reduction in muscle contractile function (3). However, patients in other studies who exhibited these characteristics presented with large scoliotic magnitudes and had undergone bracing treatment (3–5). Prolonged bracing can reduce muscle strength and lead to atrophy (28). Thus, the smaller CTS and higher fat area may result from severe scoliotic deformation and prior bracing treatment. The relationship between these factors and mild to moderate scoliosis remains unclear. Our findings fill this knowledge gap, suggesting that CST is comparable between adolescents with mild to moderate scoliosis and age-matched peers without scoliosis.

We acknowledge that the small sample size of 72 participants, including 48 AIS and 24 non-scoliosis controls, limits our interpretation of the muscle elasticity findings. However, sample size estimation and pilot study were conducted before data collection, our results satisfied statistical power to illustrate our findings. Moreover, lacking severe scoliosis with operative curve magnitude was another limitation in this study. Understanding biomechanical features of PSM in severe scoliosis helps clinicians to determine patients' spinal flexibility preoperatively (29). Additionally, the allocation of more male participants, although without significant intergroup difference, to the progression group may cause sex to be a potential confounding factor, because men and women differ in myofascial tone and their elastography findings (27). However, considering the established effects of sex on curve progression (30), we valued the opportunity to study the biomechanical characteristics of male patients with progressive AIS. Thus, we did not perform sex matching before group allocation. Moreover, this is the first study to quantify the curve progression of patients and focus specifically on the biomechanical characteristics of PSM in progressive cases. Our findings encourage diagnostic study to investigate the predictive value of PSM in scoliosis progression. Another limitation can be our use of CST to interpret muscle volume in PSM, because muscle volume can be precisely quantified using MRI. However, MRI is not a routine clinical assessment for mild to moderate AIS, and most MRI facilities are not equipped with an elastography mode for biomechanical assessment. Ultrasound elastography, as a reliable tool for characterizing the biomechanical properties of back muscles in healthy individuals and those with disease-related conditions (14), may offer an accessible and user-friendly mechanical parameter to encourage quantitative tissue assessments.

## Conclusion

Discrepancies in muscle elasticity were observed in the PSM of participants with progressive AIS who had mild to moderate curve magnitudes. Greater muscle elasticity on the concave side of the major curve was correlated with AR and more prevalent in the progressive AIS. This relationship was not detected in the CST, which did not differ between AIS and non-scoliosis controls. Our findings imply that changes in muscle elasticity occur in the early stages of scoliosis progression without changing the CST with mild to moderate AIS.

## Data Availability

The original contributions presented in the study are included in the article/Supplementary Material, further inquiries can be directed to the corresponding authors.
